# Parenting Fears and Concerns during Pregnancy: A Qualitative Survey

**DOI:** 10.3390/nursrep11040082

**Published:** 2021-11-07

**Authors:** Alisa Wilska, Anja Rantanen, Elina Botha, Katja Joronen

**Affiliations:** 1 Unit of Health Sciences, Tampere University, 33014 Tampere, Finland; alisa.wilska@gmail.com (A.W.); anja.rantanen@tuni.fi (A.R.); elina.botha@tuni.fi (E.B.); 2 School of Social Services and Health Care, Tampere University of Applied Sciences, 33520 Tampere, Finland; 3 Department of Nursing Science, University of Turku, 20014 Turku, Finland

**Keywords:** fears, motherhood, parenthood, pregnancy, primiparous, mothers, multiparous

## Abstract

Previous research on the fears and anxieties of expectant mothers has focused mostly on their fears about giving birth rather than parenting. This study aims to describe mothers’ fears and concerns about parenthood during pregnancy and to examine the similarities and differences in the perspectives of primiparous and multiparous mothers. The qualitative research for this study was conducted in three postpartum units in Finland and focused on the responses to an open-ended question about parenting fears and concerns that was part of a questionnaire given to 250 mothers after they had given birth. The responses from the 128 mothers who answered this question were subject to inductive content analysis. Fears and concerns on parenthood included worries about coping with the future and everyday life with their new baby, the psychological burden of parenthood, their maternal resources and self-efficacy, meeting their baby’s needs, their baby’s health, concerns about their relationship with their partner and financial issues. Primiparous and multiparous mothers shared many of the same concerns, but some differences emerged. The findings contribute an interesting perspective to the social debate about declining birth rates and their psychosocial causes. Further studies are needed to examine the fears and concerns of younger adults, and even teens, about parenthood.

## 1. Introduction

The birth rate trend has been declining in the European Union since 2008. In 2019, the total fertility rate (TFR) was at 1.53 births per woman in the EU [1]; the global figure is 2.403. Lancet published the global TFR scenario which forecasted the TFR to be 1.66 (95% UI 1.33–2.08) in 2100. This decline will have wide-ranging social, economic and geopolitical consequences for societies [2]. Lower fertility rates and smaller younger age cohorts, in addition to people living longer, will lead to deficiencies in the workforce [3]. Continued trends in female educational attainment and access to contraception will hasten declines in fertility [2]. There is evidence that adolescence has been extended and that other interests compete with the desire to have children, especially in women under the age of 30. Furthermore, young adults have conflicting perceptions of families with children and are afraid about parenting [4]. Our interest in this study emerged from these facts, and we wanted to explore what are the fears and concerns of pregnant women related to parenting in the low birth rate era.

Mothers experience various concerns during pregnancy, ranging from mild uncertainty to strong fears and phobias. The best known is the fear of childbirth itself [5,6]. Women who experience severe fears during pregnancy face an increased risk of emotional imbalance after they have given birth, and this has a negative impact on how they interact with their child [5]. One study found that mothers who experienced anxiety before they gave birth had more negative attitudes towards motherhood and more difficulties in adjusting to parenthood [7]. Fathers also experience childbirth fears, including harm to the mother or newborn, partner pain, feelings of helplessness, lack of knowledge and fear of high-risk intervention [8].

The literature indicates that the most common concerns expressed by pregnant women related to themselves, their pregnancy, giving birth, their child’s health and well-being and parenting [9,10,11,12,13,14,15,16,17,18,19,20,21,22,23,24,25,26]. Studies have also reported that maternal worries were most intense in early and late pregnancy [9,14], when pregnancy-related anxiety was also at its highest [27].

Studies have shown that pregnant women were most concerned about whether they would be good mothers and adjust to parenthood [10,11,14,16,20,22]. Previous literature showed that pregnant women were also concerned about how they would cope with their new baby [9,16,20,23] and whether there would be room in their hearts and lives for the new child [11]. Concerns were also raised about breastfeeding [22,24] and whether mothers had the ability to raise their children well [18]. Shahoein et al. [18] reported that some mothers were so concerned about the responsibility of being a parent that they regretted getting pregnant in the first place. Studies have also indicated that other parenting-related concerns included financial issues [9,14,18,20,23], such as worries about money [9,14], their job [14,18] and housing [20]. Research evidence has also showed that some women expressed concerns about their family life, their relationship with their partner and fears about future challenges. These included concerns about their sex life after childbirth and worries about splitting up with their partner [10,13,14,18]. It should be noted that the previous studies are often embedded in the “ideology of intensive mothering”, which includes three beliefs: (1) that childrearing should be child-centered, (2) that women remain the best primary caregivers of children, and (3) that childrearing requires tremendous amount of energy, time and money [28].

Previous research indicated that some mothers were concerned about the well-being of their existing children when they had a new baby [11] and their ability to cope with another child [14,16,18]. Petersen et al. [16] found that mothers were also concerned about how the baby’s siblings would react to the new baby. Studies by Melender [13], Öhman et al. [14], Petersen et al. [16] and Cetişli et al. [24] found that mothers’ fears and anxieties about their current pregnancy were related to experiences with previous pregnancies. They reported that first-time mothers had more concerns during pregnancy than those who had previously given birth. Studies by Melender [13] and Öhman et al. [14] found that first-time mothers were more concerned with childbirth, their child’s health and coping with their new baby than existing mothers. On the other hand, women who already had other children were more concerned about issues related to their own health, hospitalization and family finances [14].

Studies on parental concerns among mothers during the post-partum period have indicated that the concerns and fears are quite similar to those experienced during pregnancy. Nomaguchi and Milkie [29] found that new mothers reported higher levels of conflict with spouses compared with their childless counterparts. Kaitz’ (2007) study identified six dimensions of maternal concerns in the period three to six months post-partum: family health, return to work, personal well-being, relationship/support, infant care and spouse. Returning to work and family health were the categories of most concern and the overall intensity of concerns decreased from the third month to the sixth month after giving birth [30].

Previous research has focused on the fears and anxieties of expectant mothers both about giving birth and about parenting. The current state of low birth rate trends warrants updated research on new mothers’ concerns about becoming parents with a view to tackling those fears. We also need to know more about the concerns that pregnant women are expressing during their pregnancy in order to introduce these topics into the discussion with professionals in the maternity services. This study investigated expectant mothers’ fears and concerns about becoming a parent during the first days of the postpartum period which, to our knowledge, have received relatively little attention from researchers. The aim was to inductively describe current mothers’ fears and concerns about parenting while they were pregnant and to deepen our understanding of the factors that caused their anxieties. We also wanted to examine any possible differences, and similarities, between primiparous and multiparous mothers.

## 2. Materials and Methods

### 2.1. Procedure and Participants

A cross-sectional study was conducted on a convenience sample of 250 primiparous and multiparous mothers who were in three different postpartum units of one university hospital from 1 March to 20 May 2019. This paper reports the findings of a subsample (*n* = 128 mothers) derived from a primary sample of 250 mothers. The mothers in the primary sample who answered the open-ended question about fears and concerns about parenting during their pregnancy were included in this study; the response rate was 51.2%. Just over half of these 128 respondents were multiparous mothers (50.8%), compared to 51.2% in the main sample, which was in line with the primary sample. The mean age of the 250 mothers in the primary sample was 30.5 years ± 4.78 (range 19–44) and 95.6% were married or cohabiting. Almost half had received secondary, upper secondary or vocational education (48.8%) and just over half (51.2%) had a university degree. Before their maternity leave, 64.4% were employed full time and the rest were employed part-time or unemployed.

This Finnish university hospital handles about 4400 deliveries a year and the postpartum units care for mother-infant dyads with no health problems for an average of 1–3 days.

The participants were recruited by the midwives working in the three units, as part of a larger mixed-methods study. All the mothers who fulfilled the inclusion criteria were invited to take part. They were included if they had delivered single infants who were healthy and rooming in with their mothers. We excluded multiple births and mothers who were unable to understand Finnish. The principal researcher visited the postpartum units to speak to the midwives about the study, both before and during the data collection period.

All mothers (*n* = 250) who agreed to participate were asked to complete the questionnaire independently at the hospital before discharge. 

### 2.2. Qualitative Survey

The research material was collected as part of a larger mixed-methods study on mothers’ self-efficacy and parenting satisfaction during the postpartum period. The data were collected 1–3 days after birth and the mothers were asked to complete a questionnaire that comprised 72 structured questions and two open-ended questions. This study focuses on the responses to one of the open-ended questions, which asked the mothers if they had experienced any fears and concerns about parenting during their pregnancy. The question was: “*If you had fears or worries concerning parenthood during the pregnancy, what were you afraid of or worried about?*” The results that emerged from these data were not possible to report in the previous paper due to its different perspective from the mothers’ self-efficacy and parenting satisfaction. Qualitative survey questions provide the opportunity to gather a diversity of perspectives and experiences, especially when the population of interest is large and diverse [31].

The mothers filled in the printed questionnaire during their hospital stay in the privacy of their rooms. The questionnaires were then handed to one of the midwives in a closed envelope and locked in the study box that had been provided. The principal researcher collected the questionnaires, which were completed once a week.

The findings of the structured section of the questionnaire have previously been reported, along with the demographic data of the respondents [18].

### 2.3. Data Analysis

The data were analyzed from three perspectives. The first was that of the fears and concerns shared by all the mothers. The second and third perspectives focused separately on the primiparous and multiparous groups.

The research material was analyzed by inductive content analysis for each of the three perspectives. This is a method that makes it possible to distil words into fewer content-related categories [32]. Before the analysis, the first author (AW) carefully read the written research material several times to obtain a sense of the whole results. The meaning units that were analyzed were the phrases, sentences and words, and only clearly expressed content and comments relevant to the research question were included [33].

The analysis was guided by the research task. First, all the meaning units were numbered and color-coded; this showed that they were relatively concise and informative. These were then reduced down to 244 condensed meaning units [33].

Thirdly, the condensed meaning units were grouped together by their similarities and differences and 43 sub-categories were formed. Sub-categories were named with abstract topics that described the content of the condensed meaning units. The abstraction process continued and 13 categories were created based on classification and abstraction. These were then grouped into three main categories, which were: (1) fears and concerns shared by both groups; (2) fears and concerns just expressed by first-time mothers; (3) fears and concerns just expressed by mothers who already had children. The final decision about the sub-categories, categories and main categories was made during discussions with the whole research team. An example of how the analysis progressed is presented in Table 1. This followed the guidance provided by Elo and Kyngäs [32] on content.

### 2.4. Ethical Considerations

The Regional Ethics Committee of the University Hospital approved the study protocol (R18188H), which was consistent with the 1975 revision of the Declaration of Helsinki. The hospital administrators also provided their permission for the study. The data were collected during from 1 March to 20 May 2019 and were protected in line with the European Union’s General Data Protection Regulation 2016/679 [34]. The mothers received written and verbal information about the study and provided signed, informed consent.

## 3. Results

Maternal fears and concerns related to parenthood during pregnancy were divided into those that were shared by both groups of mothers and those that were just expressed by primiparous or multiparous mothers. The findings are presented in Table 2.

### 3.1. Fears and Concerns Shared by Both Groups

When they were pregnant, both groups worried about coping with the future and everyday life with a baby, the psychological distress, their maternal resources, parental self-efficacy, meeting their baby’s needs, their baby’s health and well-being, their relationships with their partner and their financial situation.

Coping with the future included fears about the challenges they would face, including safeguarding their child’s future.

*“Can one of my small choices ruin the entire life of my child?” (Mary, primipara).*

Both groups expressed concerns about coping with everyday life. This comprised time management, giving up their own time, practical arrangements, being woken up at night, insomnia, loneliness and lack of a support network with their new baby. Mothers worried about how they would be able to organize things so that there would be enough time for everything. Some mothers also expressed concerns that they would experience sleep problems when their baby woke up during the night.

*“I’m afraid I’ll never get a good night’s sleep again” (Tina, primipara).*

A few of the mothers were also worried about being alone and feeling lonely at home with the baby. Some expressed fears that their existing support network was inadequate or that they lacked close networks.

While they were pregnant, the mothers were concerned about the psychological distress they would experience once their baby was born. This included worries about the mental health burden, changes in their identity and postpartum depression. They also worried whether they would have enough psychological resources after the birth of their baby and were frightened that their mental well-being would be jeopardized by the mental health burden and identity changes. Some mothers expressed concerns about postpartum depression, especially if they had suffered from it after a previous birth.

*“My main concern was the recurrence of postpartum depression” (Sally, multipara).*

In terms of maternal resources, respondents were concerned about their own health, coping, fatigue and whether they would be a good enough mother.

*“I was also worried about how I would cope after the baby was born, because I was very tired throughout my pregnancy” (Britney, multipara).*

In addition to coping and protecting their well-being, pregnant mothers were concerned about their parental self-efficacy and their ability to meet their baby’s needs. They were also worried about their baby’s health and well-being. In terms of parental self-efficacy, mothers were worried about the responsibility of being a mother and whether they would be a successful mother. Other concerns were their inadequate knowledge of parenting and inadequate parenting skills.

*“I had concerns about how to be a good mother” (Cindy, primipara).*

*“I had probably the same thoughts as all expectant mothers about how to survive” (Helen, primipara).*

Both groups were concerned about the responsibility of motherhood.

*“I worried about how I can guarantee a safe life and environment for my child” (Camilla, multipara).*

Pregnant mothers’ concerns about whether they could meet their baby’s needs included fears about their ability to create an emotional bond with their child. They were also worried about handling their baby, ensuring their baby slept well and breastfeeding.

*I was worried about “whether breastfeeding succeeds, whether the feelings of love will awake for the child, whether I can properly take care of the baby” (Emily, primipara).*

Pregnant mothers were concerned about their baby’s health and well-being and whether they would get sick and display fussy behavior.

*“The child’s health was a bit of a concern, although there was no specific concern” (Jasmin, primipara).*

The mothers also expressed concerns about their relationship with their partner after they gave birth. This included worries about how their partners would cope and maintain their own health and about their general and sexual relationship.

*“How to find time together with my spouse after the birth of the baby” (Barbara, primipara).*

Mothers also worried about their financial situation, including changes in their economic status and whether they would have enough money.

### 3.2. Fears and Concerns Just Expressed by First-Time Mothers

The first-time mothers expressed two specific concerns that were not mentioned by the women who already had children. These related to wide-ranging and comprehensive changes in their lives when they became a parent and intrusive outsiders.

*“I was a little worried about everything” (Emma, primipara).*

Fears about intrusive outsiders included outsiders criticizing them and judging their parenting skills and educational advice from third parties.

*“Intrusive advice in terms of value education” (Linda, primipara).*

### 3.3. Fears and Concerns Just Expressed by Mothers Who Already Had Children

Mothers who already had children at home expressed two fears about parenting that first-time mothers did not have. Their greatest fears were how their other children would react to their new baby, especially if they were very young. A small age gap between the children worried the mothers as well. They also expressed concerns about how they would cope with several children. One mother who already had a child worried about “how my firstborn would react to the baby” (*Eva, multipara*).

These mothers were also worried about making sure they gave their existing children enough attention and creating an emotional bond with their new baby.

*“Can I love both children equally and treat them that way too?” (Sophia, multipara).*

The challenge of looking after their new baby and existing children was also a concern. Mothers were concerned about everyday life with the larger family. They also feared for their own survival and resilience with more children.

*“How do I manage to care properly for my baby when I am also caring for a lively three-year-old?” (Penelope, multipara).*

*“How do I cope with three small children?” (Nora, multipara).*

## 4. Discussion

The results of this study show that pregnant women had a number of fears and concerns about future parenting. Some were shared by both first-time and existing mothers, while some were unique to each group. To our knowledge this was the first large-scale qualitative survey to gather data on parenting fears inductively by asking both primiparous mothers and multiparous mothers to respond to an open-ended question. Our findings partly support the results of previous studies, but they add extra information on the fears and concerns that expectant mothers experienced about parenting. The findings broaden the knowledge and understanding of this phenomenon.

The first interesting result was that the mothers in our study shared very similar concerns about parenting. This was in contrast to previous studies that reported both quantitative and substantive differences between first-time mothers and women who already had children [13,14,16,24]. Studies by Melender [13] and Öhman et al. [14] found that first-time were more concerned about childbirth, child health and coping with their baby than mothers with other children. In this study, both groups expressed worries about their ability to meet their baby’s needs and their baby’s health and well-being. However, only first-time mothers expressed fears about comprehensive changes in their lives and intrusive outsiders.

This study also indicated that mothers with existing children were concerned about older siblings and how to cope with several children, which was obviously not a concern for the first-time mothers. These results were in line with previous studies, which also found that mothers were worried about how their existing children would react to their new baby and how it would change their relationships with them [11]. The existing mothers in our study were also worried that they would not be able to satisfy the needs of their older children [18].

When it came to childcare, the mothers in our study were concerned about breastfeeding, regardless of how many babies they had delivered. This reflected earlier research by Dornelles et al. [22] and Cetişli et al. [24] showing that pregnant women feared they would be unable to breastfeed their baby. However, breastfeeding was just one of the 13 different concerns that the mothers in our study expressed. It is crucial to underline the fact that pregnant mothers need support with several parenting issues, such as parental self-efficacy and coping with everyday life with their new baby. This support is needed regardless of whether the woman is expecting her first baby or already has children. Global baby-friendly programs that support breastfeeding, including those provided by the World Health Organization [35], are of great value. However, the results of this study suggest that mothers also need support with many other parenting skills and that these should be addressed by baby-friendly programs. The results of this study can be used also in maternity and child health services which offer a natural forum to discuss the fears and concerns with professionals.

It is surprising that the mothers in our study did not address fears or concerns of their co-parent. This may be due to the question we asked, namely, “During the pregnancy, what were *you* afraid of or worried about concerning parenthood?” In Finnish the word *you* (sinä) refers to the second person singular. Mothers however expressed concerns about their relationship with their partner after they gave birth.

## 5. Strengths and Limitations

The main strength of this study was the diverse dataset from a relatively large number of mothers. This made it possible to draw a larger picture about phenomena that have mostly been investigated by structured questionnaires or qualitative methods with small samples. A wide-angle lens also allowed us to identify fears and concerns about parenting from *different* groups, i.e., primiparas and multiparas within a wider population of pregnant women [31]. In addition, the women were surveyed 1−3 days after giving birth and this minimized the issues normally associated with studies that rely on recall.

The survey design embodied a participant-centred research practice, allowing participants control over their research participation. Newborn mothers with caregiving obligations require such flexibility in surveys in order to participate [31].

This study had some limitations. First, the fears and concerns were explored by one open-ended question, and only about the half of the primary sample of 250 mothers responded to the question. It is not possible to determine whether the mothers who responded to the question had more or fewer concerns than those mothers who did not respond. However, the proportions of first-time mothers and those who already had children were almost the same in the whole group of 250 mothers and in the 128 who were included in this study. Second, the responses to the question were quite short, which meant that a more extensive qualitative analysis was not possible. Third, one disadvantage of this study is that participation in the survey requires literacy skills, so that mothers with limited literacy skills might refuse to participate in this study.

## 6. Conclusions

Pregnant mothers said that they had a wide range of fears about parenting when they were pregnant, and these concerns varied from individual to individual. First-time mothers and those who already had children shared many of the same fears and concerns. These were also some worries that were unique to each of the two groups. For example, mothers who had given birth before were worried about how their new baby’s siblings would react. The study provides comprehensive inductive descriptions, as well as new insights, into the fears and concerns that pregnant women had about parenting. The findings also contribute an interesting perspective to the social debate about declining birth rates and their psychosocial causes, such as pregnant women’s overwhelming worries about themselves, the baby’s well-being and life with the new baby. With that in mind, we feel that it is important to carry out research examining the fears and concerns of younger adults, and even teens, about parenthood. This should be carried out before they actually plan to start a family.

## Figures and Tables

**Table 1:** An example of how the data were condensed and abstracted, using the shared fears and concerns expressed by both primiparous and multiparous mothers.

Condensed Meaning Unit ^1^	Sub-Category	Category
Child’s general health, with no specific reason (4, P)	Baby’s health	Baby’s health and well-being
Child’s health (4, 22, 42, 50, 60, 63, P; 67, M)		
Issues related to the baby’s health (28, P) Baby’s overall health (65, 80, 86, P; 67, 82, M) Baby’s health although everything was okay (82, M) Whether baby is healthy (99, M)		
Baby getting sick (6, P)	Baby’s getting sick	
Baby having problems with bowel function (11, P) Baby experiencing pain (v11, P)		
Worrying about the baby (32, P)	Baby’s well-being	
Worried about sudden infant death syndrome (96, P) Worrying about something happening to the baby (109, P) Worried something bad will happen to the baby (119, P)		
Baby displaying fussy behavior (6, P)	Baby’s fussiness	
Baby crying (11, P)		

^1^ Mother’s participant number. M = multiparous; P = primiparous.

**Table 2:** Maternal fears and concerns related to parenthood during pregnancy.

Main Category	Category	Sub-Category
Fears and concerns shared by both groups	Coping with the future	Challenges of future
		Safeguarging child’s future
	Coping with everyday life with a baby	Time management
		Giving up their own time
		Practical arrangements
		Being woken up at night
		Insomnia
		Loneliness
		Lack of a supportive network
	Psychological distress	Mental burden
		Changes in their identity
		Postpartum depression
	Maternal resources	Mother’s own health
		Mother’s coping
		Mother’s fatigue
		Being a good enough mother
	Parental self-efficacy	Responsibility of being a mother
		Being a successful mother
		Inadequate knowledge of parenting
		Inadequate skills of parenting
	Meeting their baby’s needs	Ability to create an emotional bond with their child
		Handling the baby
		Ensuring their baby slept well
		Breastfeeding
	Baby’s health and well-being	Baby health
		Baby well-being
		Baby’s getting sick
		Baby’s fussy behavior
	Relationship with the partner	Coping of the spouse
		Health of the spouse
		General relationship with the spouse
		Sexual relationship with the spouse
	Financial situation	Changes in economic status
		Money and its sufficiency
Fears and concerns just expressed by first-time mothers	Wide-ranging and comprehensive changes	Worry about everything
		Life change
	Intrusive outsiders	Criticism of the outsiders
		Educational advice from third parties
Fears and concerns just expressed by mothers who already had children	Other children’s reactions to the new baby	Small age gap between the children
		Siblings’ reactions
	Coping with several children	Sharing attention
		Creating an emotional bond with the new baby

## Data Availability

According to the ethical statement of this study, the data are not publicly available.
